# Supplementary material for: Medium Bandgap Polymers for Efficient Non-Fullerene Polymer Solar Cells—An In-Depth Study of Structural Diversity of Polymer Structure
Source: Int J Mol Sci. 2022 Dec 28;24(1):522. doi: 10.3390/ijms24010522 (PMC9820695; doi:10.3390/ijms24010522)
Supplement: Supplementary file 1 [file ijms-24-00522-s001.zip › ijms-2105338-supplementary.pdf]

Supplementary Materials

Medium Bandgap Polymers for Efficient Non-Fullerene Polymer Solar Cells—An In-Depth Study of Structural Diversity of Polymer Structure

Shimiao Zhang [1], Dong Hwan Son [2,3], Rahmatia Fitri Binti Nasrun [2,3], Sabrina Aufar Salma [2,3], Hongsuk Suh [1,*] and Joo Hyun Kim [2,3,*]

[1] Department of Chemistry and Chemistry Institute for Functional Materials, Pusan National University (PNU), Busan 46241, Republic of Korea
[2] CECS Research Institute, Core Research Institute, Busan 48513, Republic of Korea
[3] Department of Polymer Engineering, Pukyong National University, Busan 48513, Republic of Korea
* Correspondence: hssuh@pusan.ac.kr (H.S.); jkim@pknu.ac.kr (J.H.K.)

Keywords: non-fullerene acceptor; indandione; polymer solar cell; thiophene bridges; inverted solar cell

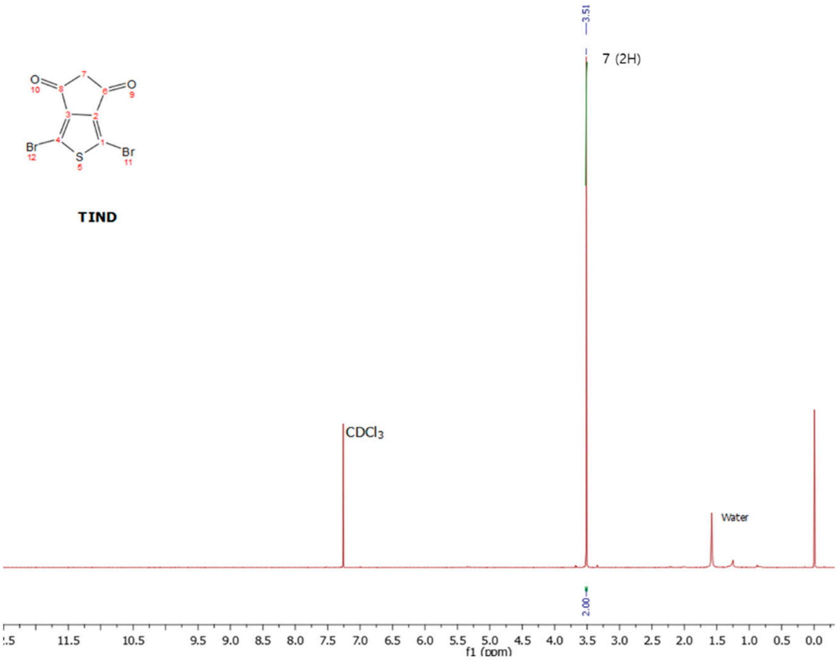

Figure S1. (^{1}H NMR spectrum of compound **IND**).

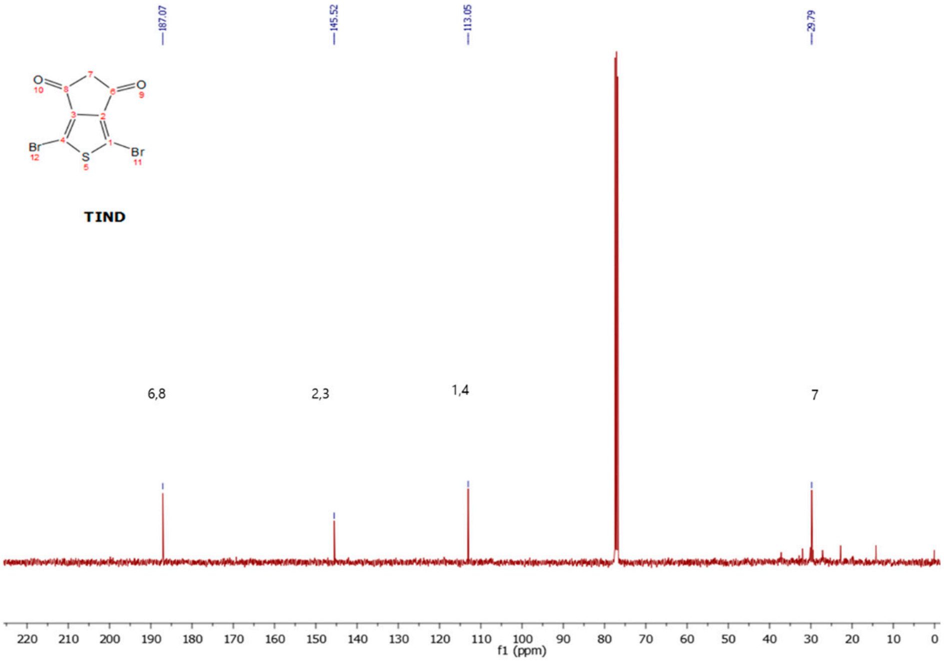

Figure S2. (^{13}C NMR spectrum of compound **IND**).

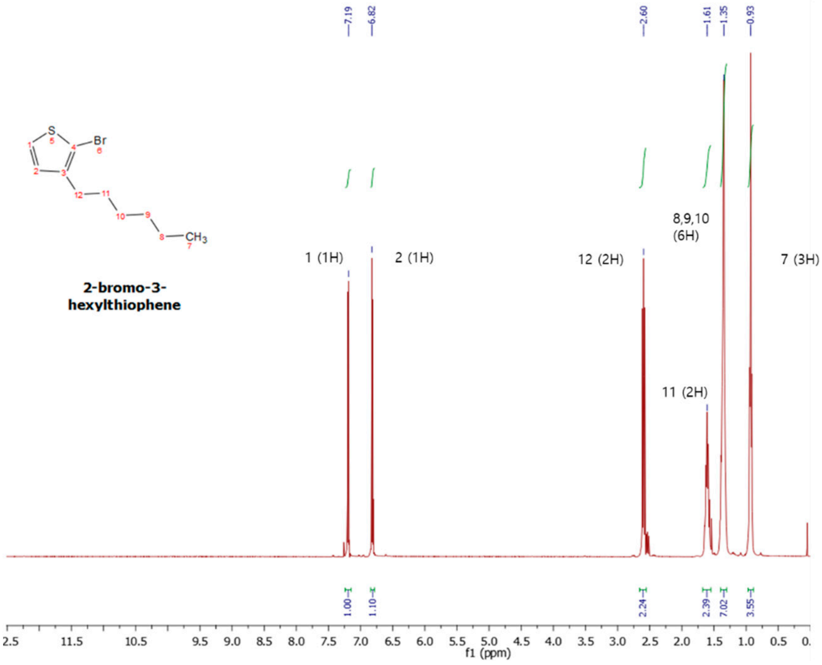

Figure S3. (^{1}H NMR spectrum of compound **2**).

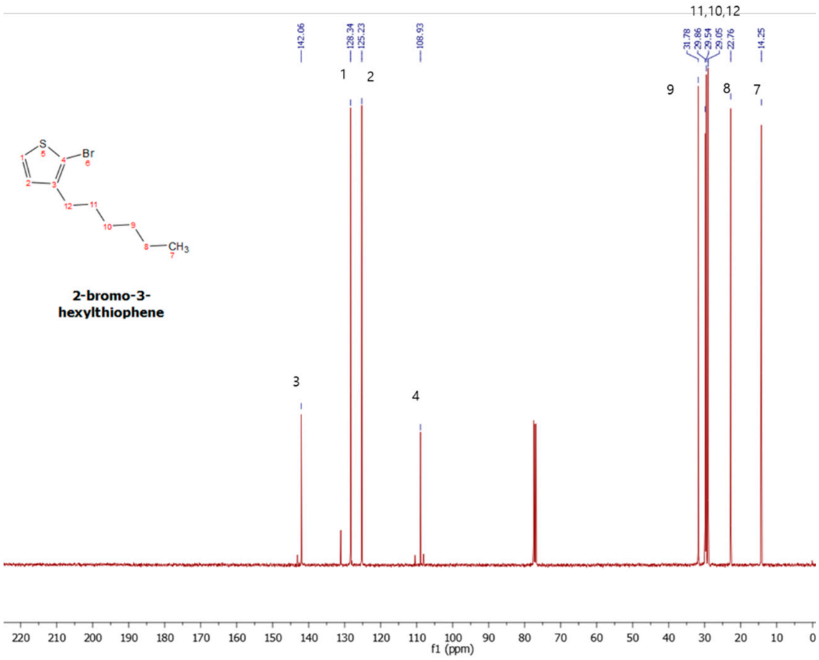

Figure S4. (^{13}C NMR spectrum of compound **2**).

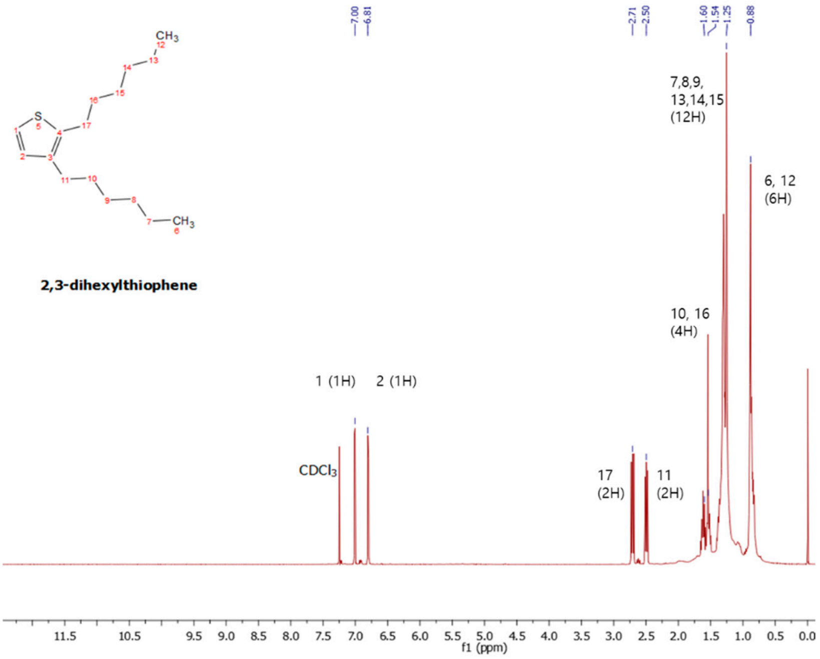

Figure S5. (^{1}H NMR spectrum of compound **3**).

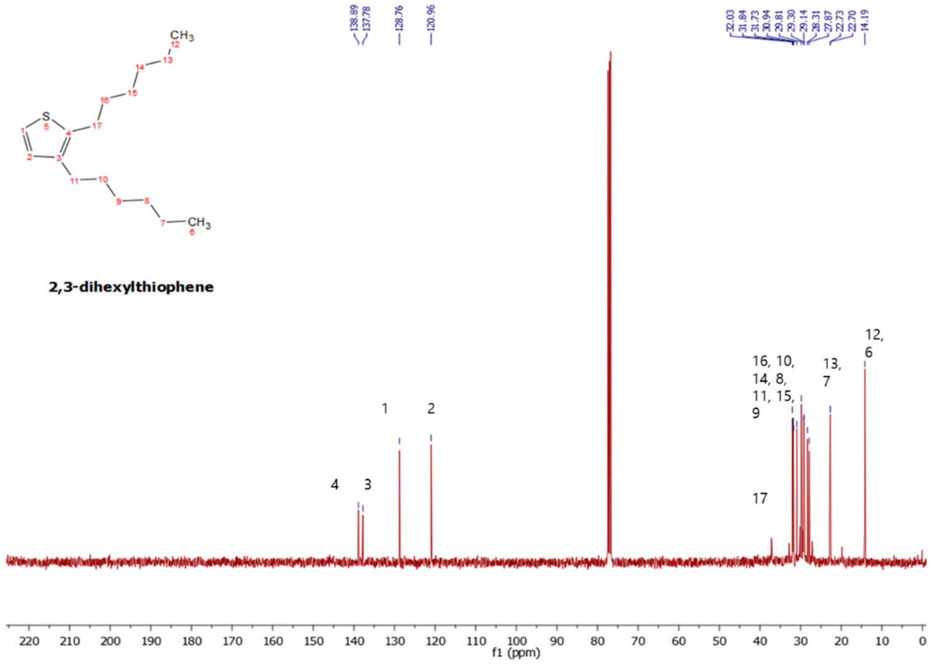

Figure S6. (^{13}C NMR spectrum of compound **3**).

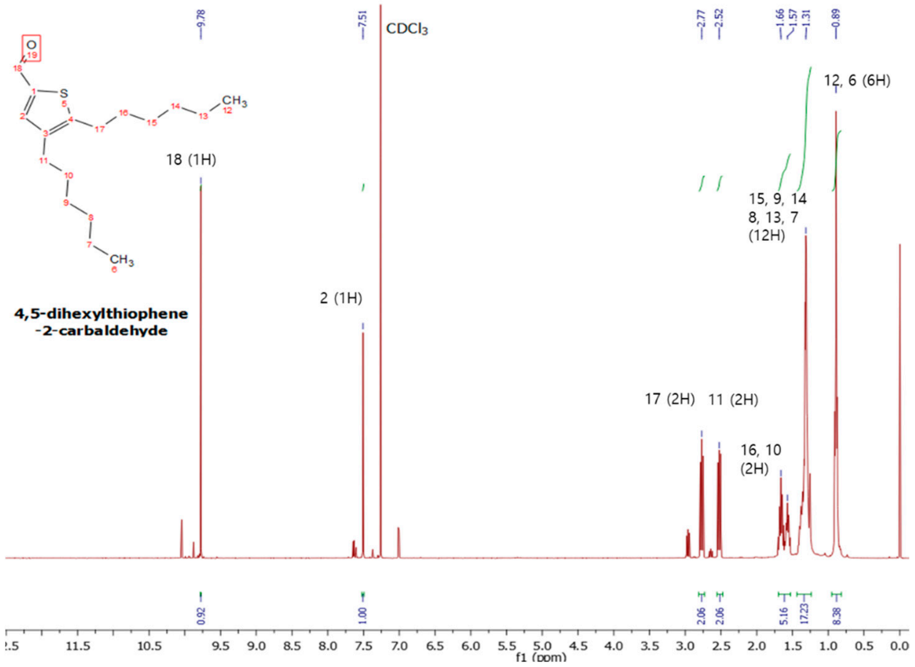

Figure S7. (^{1}H NMR spectrum of compound **4**).

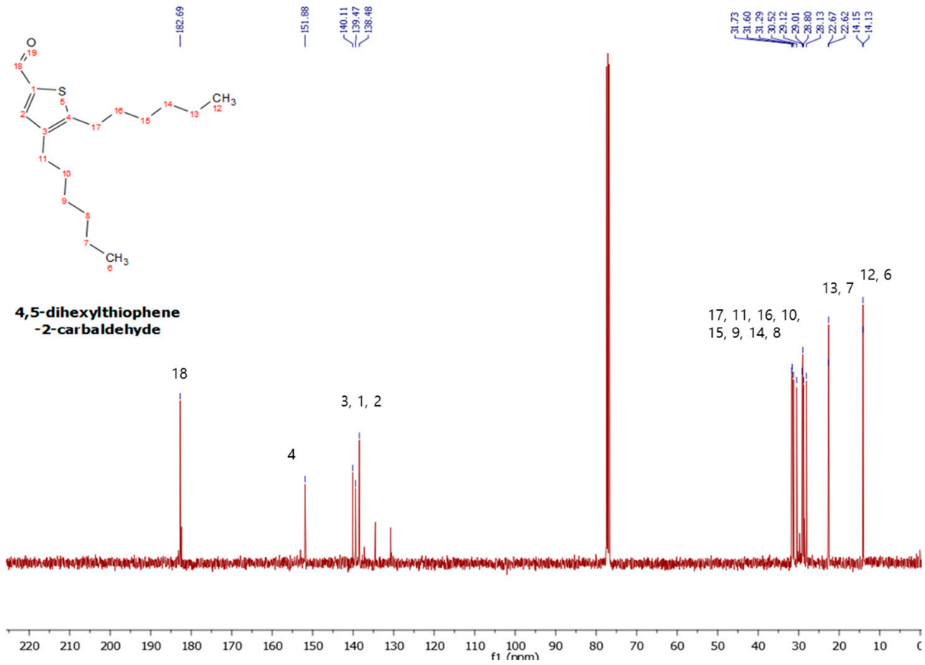

Figure S8. (^{13}C NMR spectrum of compound **4**).

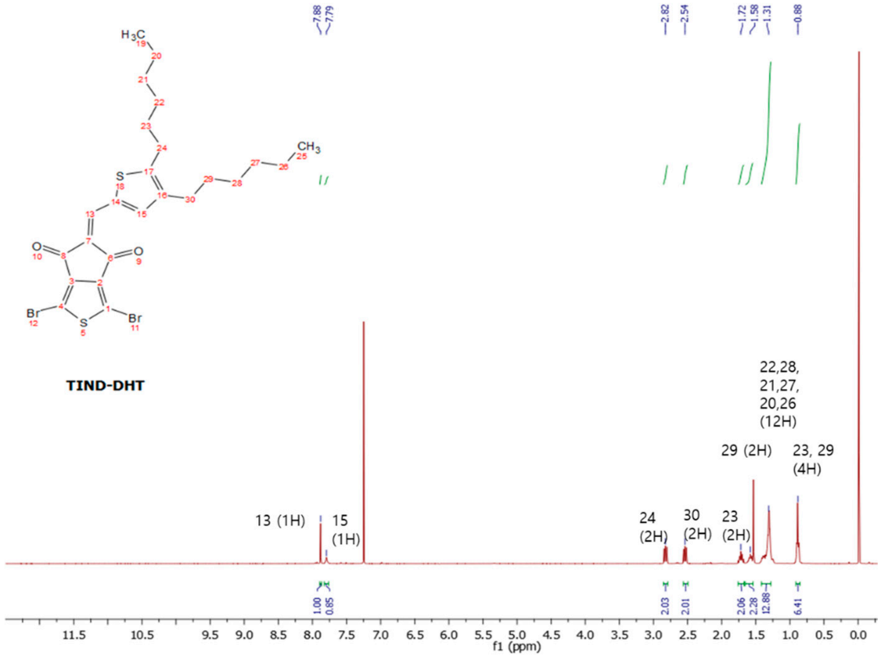

Figure S9. (^{1}H NMR spectrum of compound **5**).

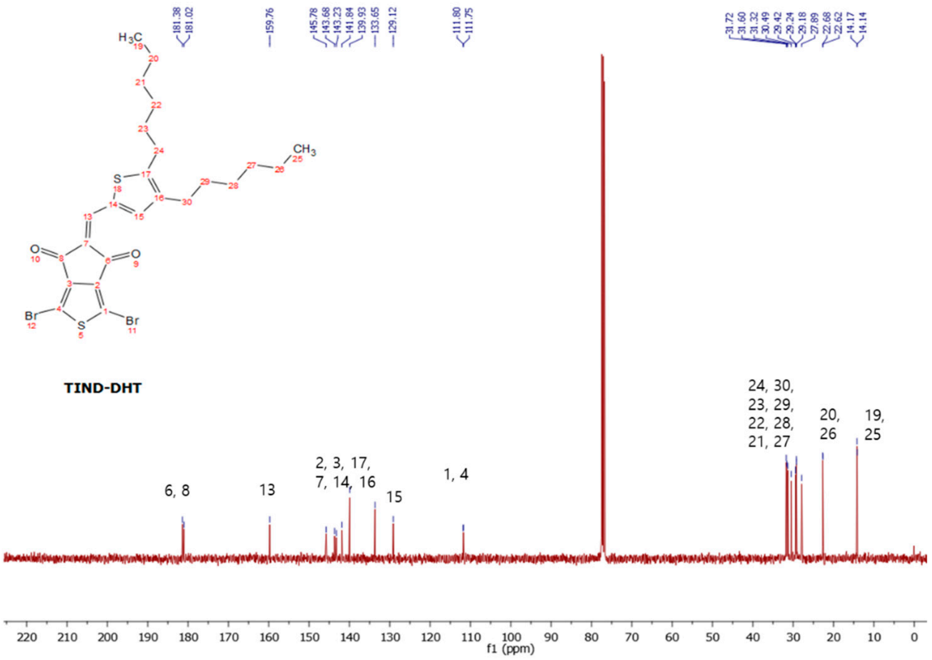

Figure S10. (^{13}C NMR spectrum of compound **5**).

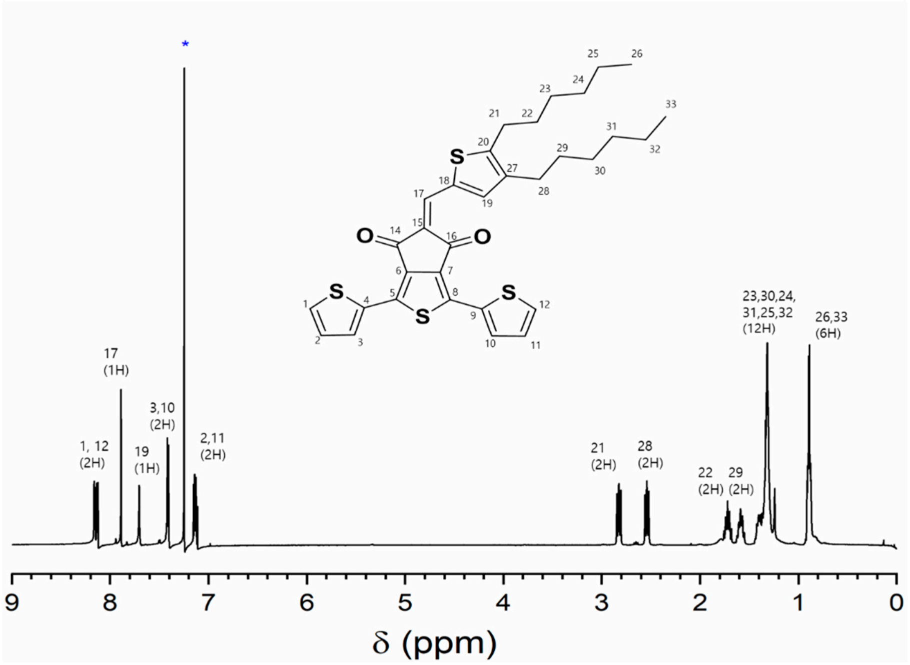

Figure S11. (^{1}H NMR spectrum of compound **6**).

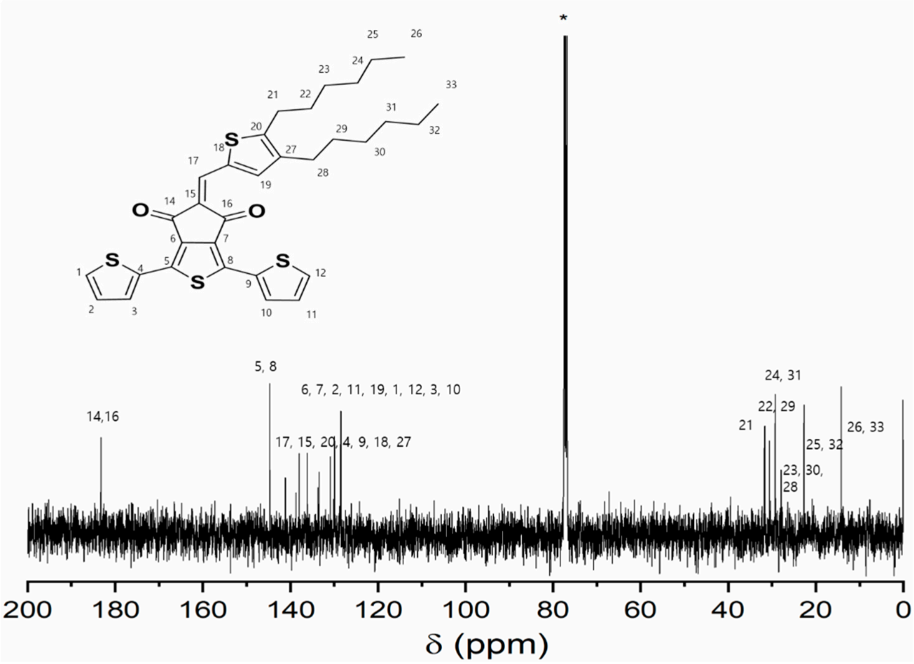

Figure S12. (^{13}C NMR spectrum of compound **6**).

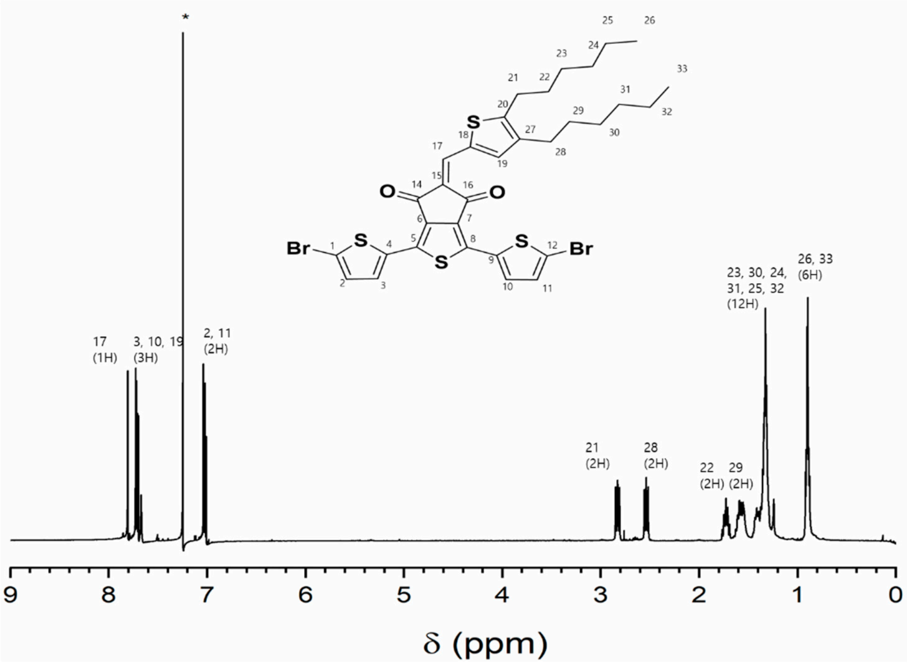

Figure S13. (^{1}H NMR spectrum of compound **M1**).

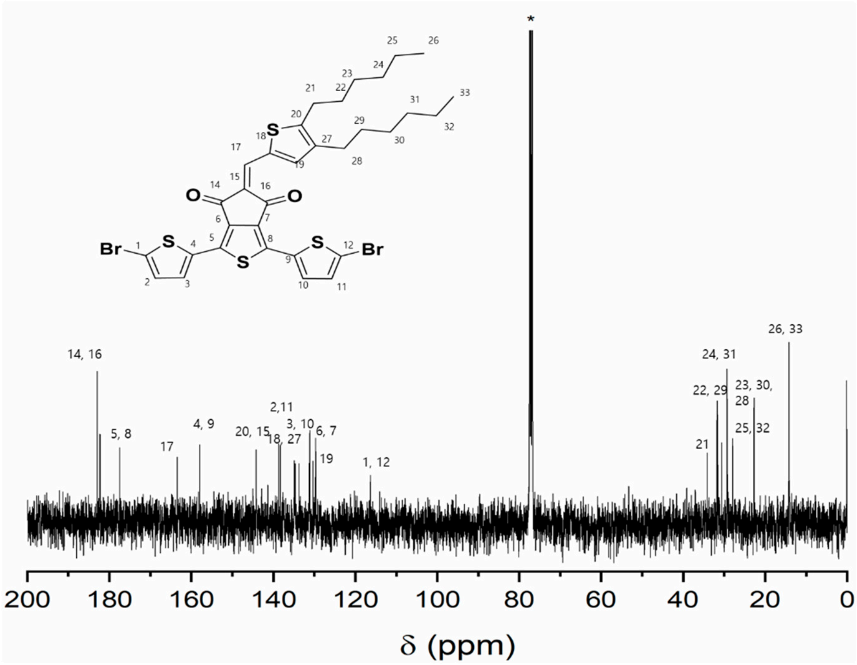

Figure S14. (^{13}C NMR spectrum of compound **M1**).

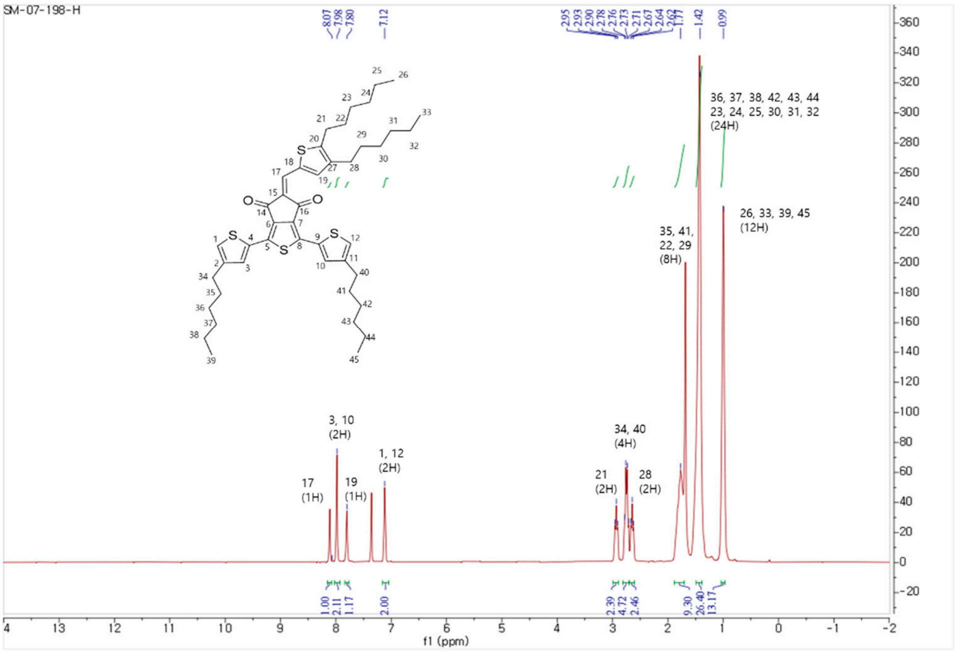

Figure S15. (^{1}H NMR spectrum of compound **7**).

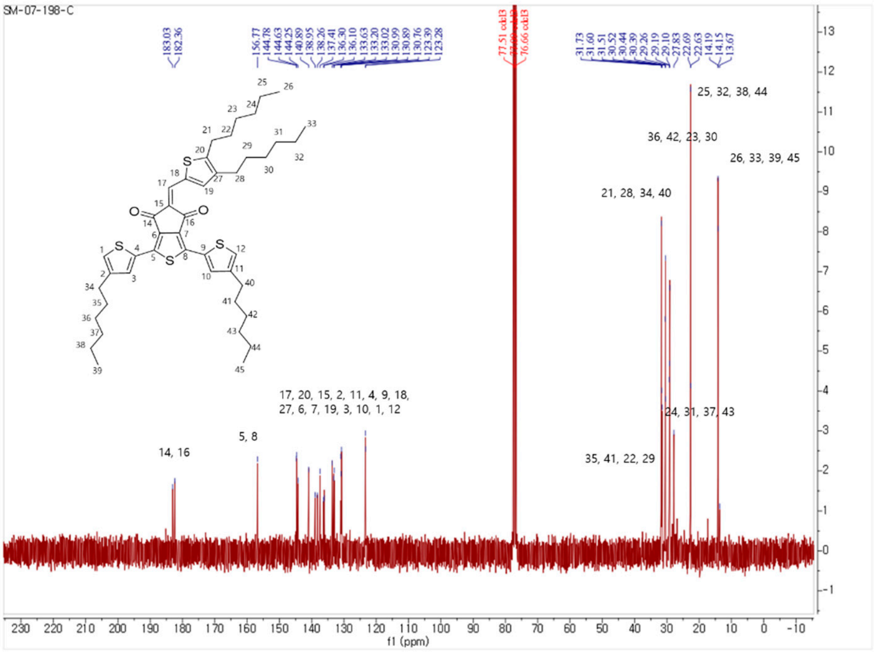

Figure S16. (^{13}C NMR spectrum of compound **7**).

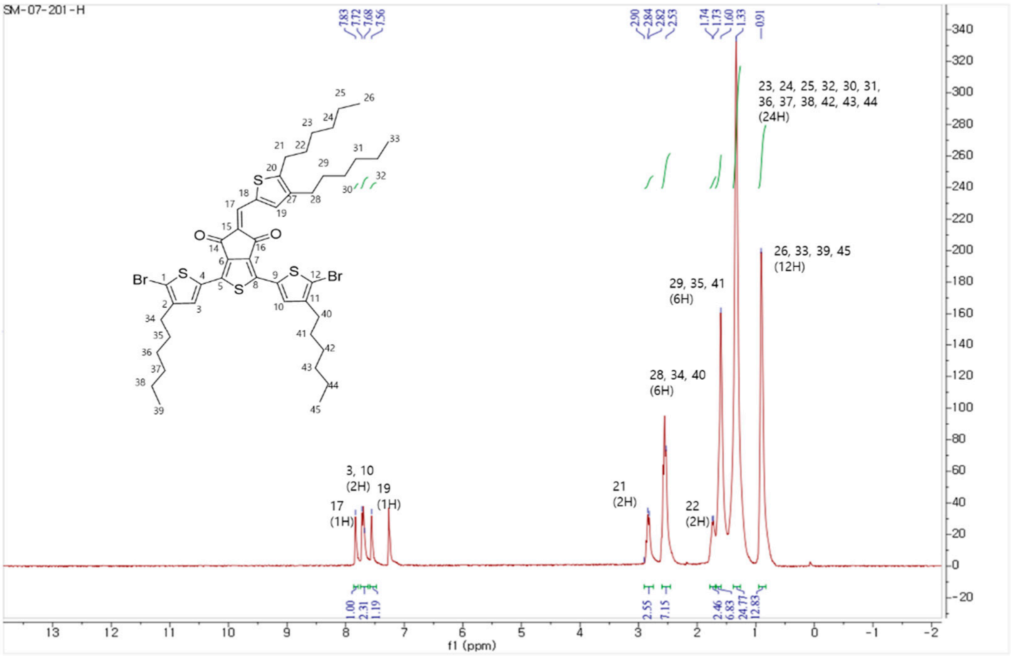

Figure S17. (^{1}H NMR spectrum of compound **M2**).

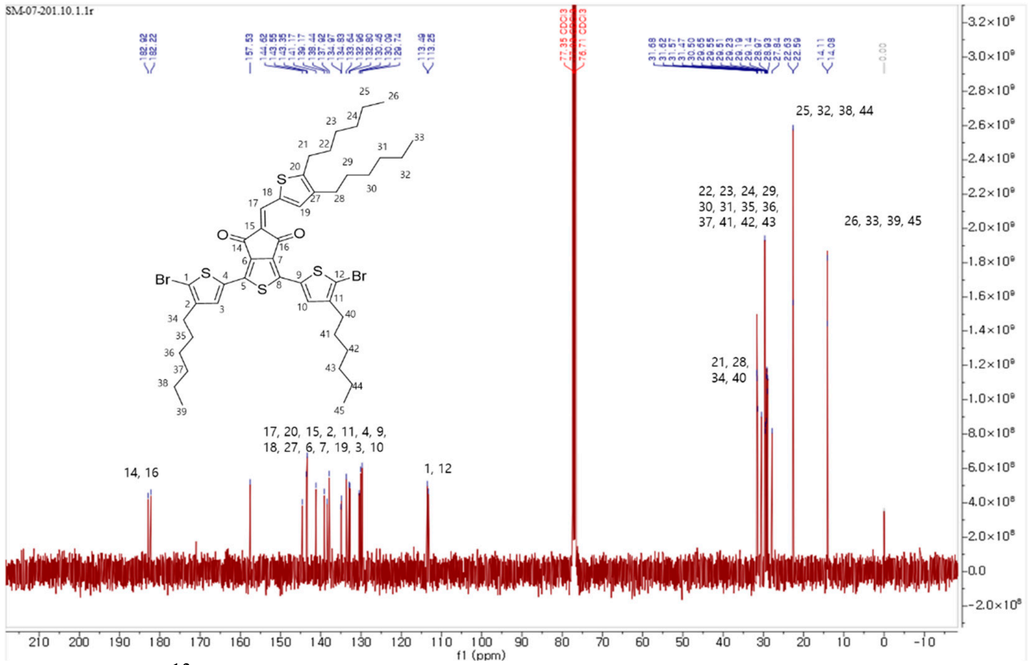

Figure S18. (^{13}C NMR spectrum of compound **M2**).

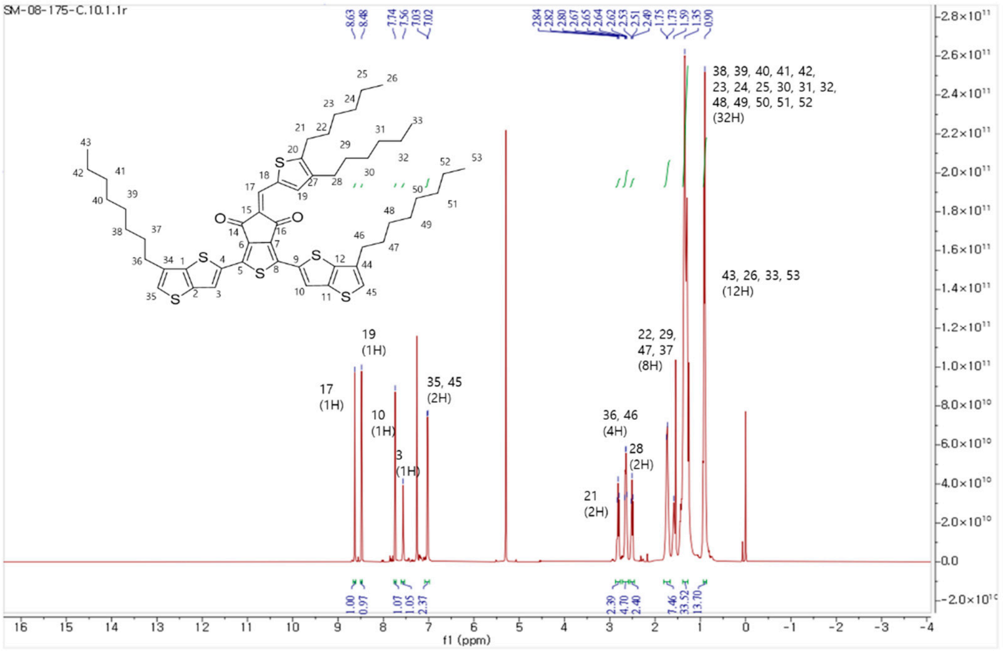

Figure S19. (^{1}H NMR spectrum of compound **8**).

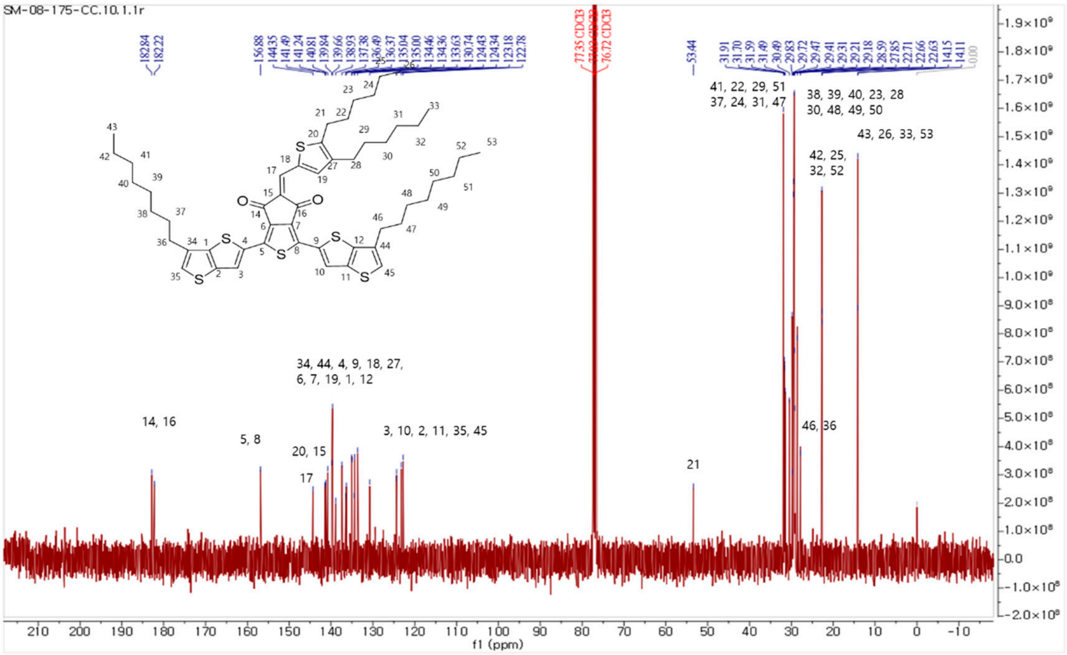

Figure S20. (^{13}C NMR spectrum of compound **8**).

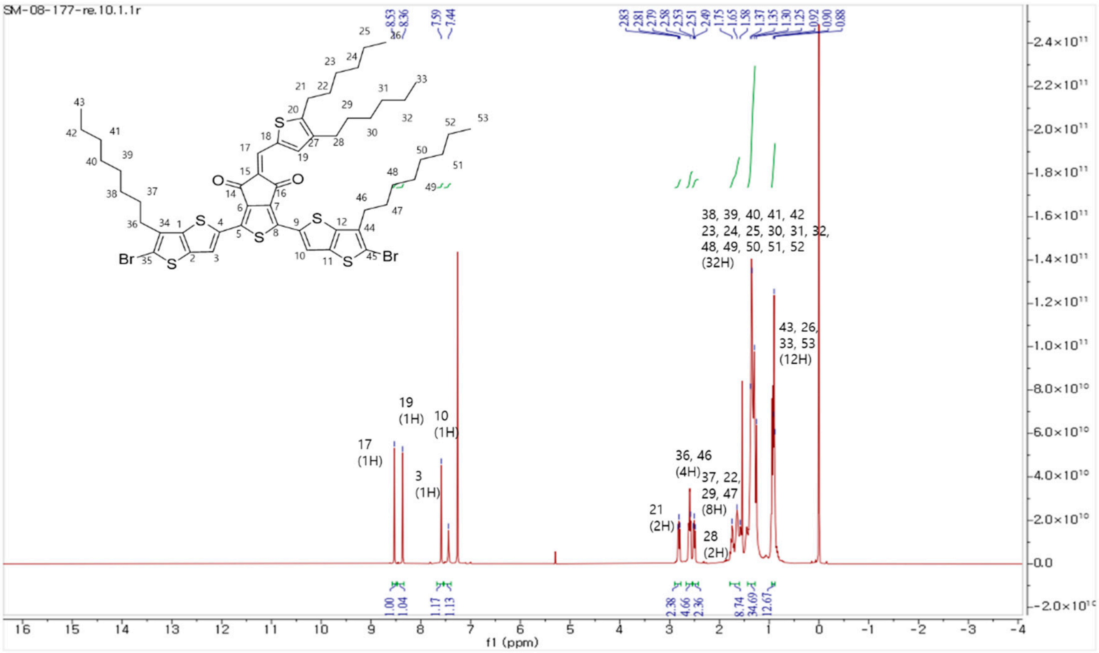

Figure S21. (^{1}H NMR spectrum of compound **M3**).

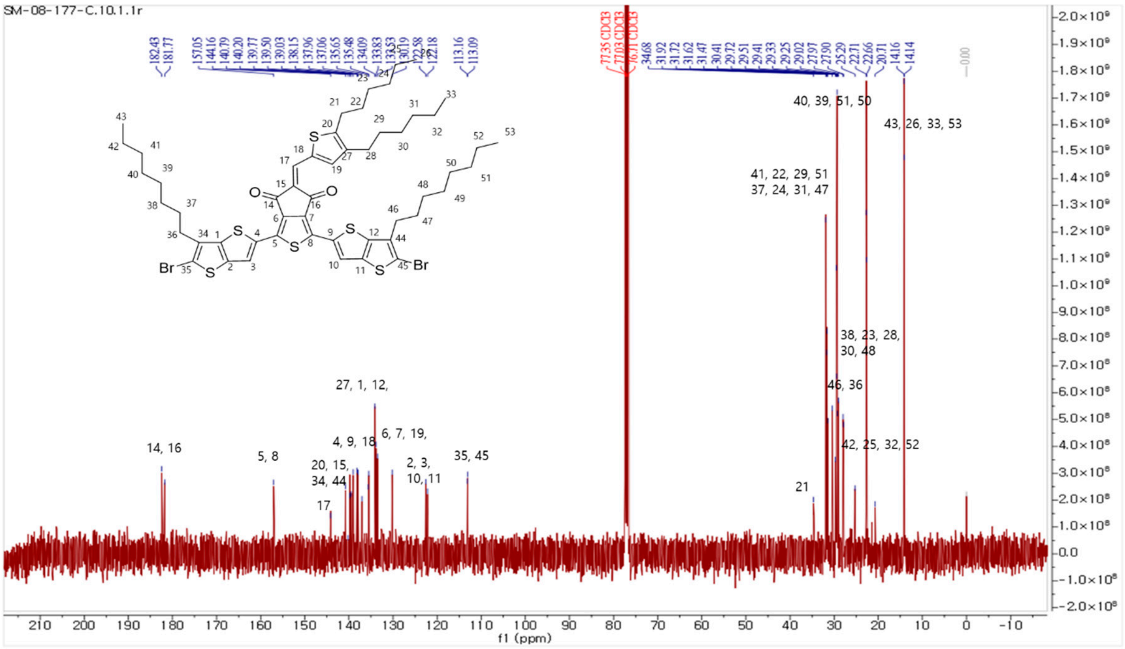

Figure S22. (^{13}C NMR spectrum of compound **M3**).

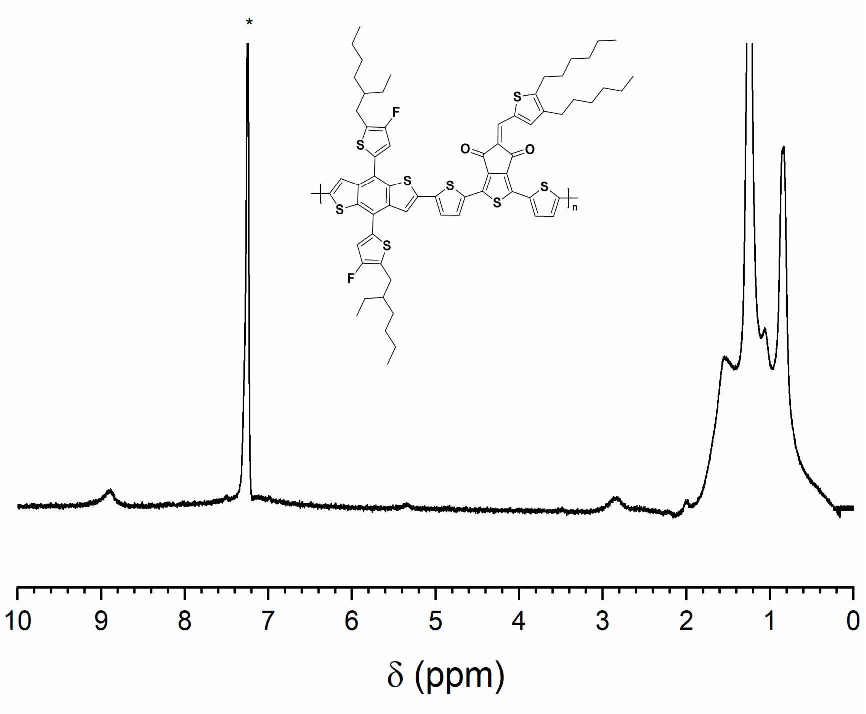

Figure S23. (^{1}H NMR spectrum of **IND-T-BDTF**).

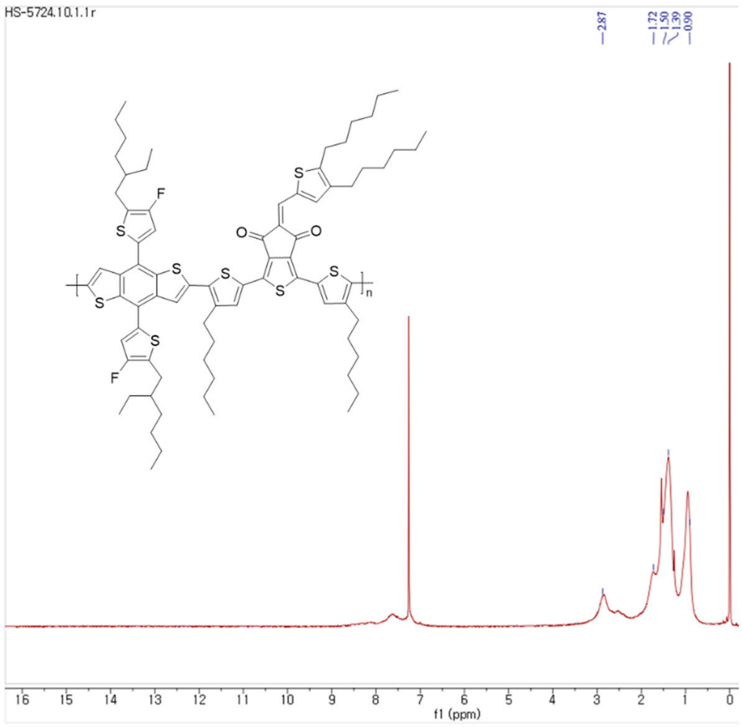

Figure S24. (^{1}H NMR spectrum of **IND-HT-BDTF**).

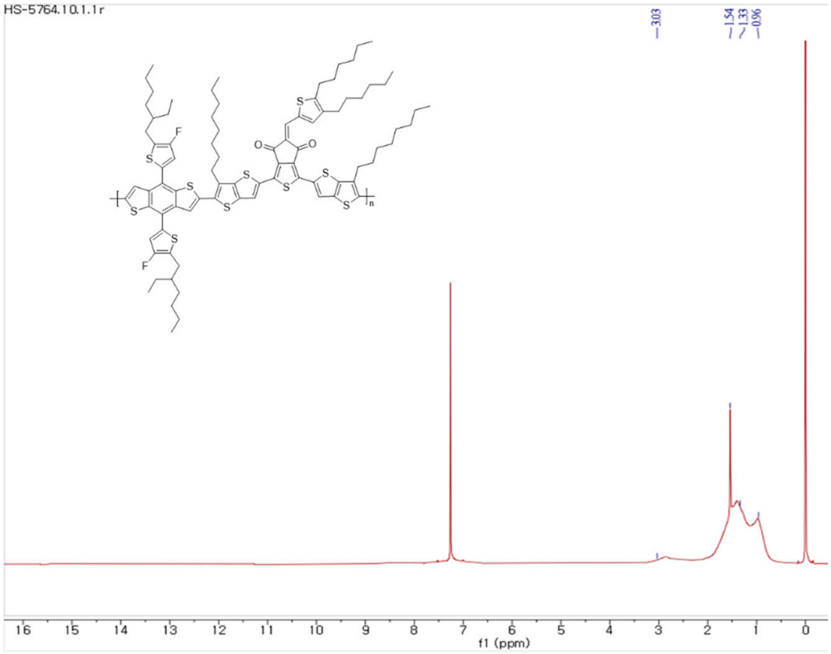

Figure S25. (^{1}H NMR spectrum of **IND-OTT-BDTF**).

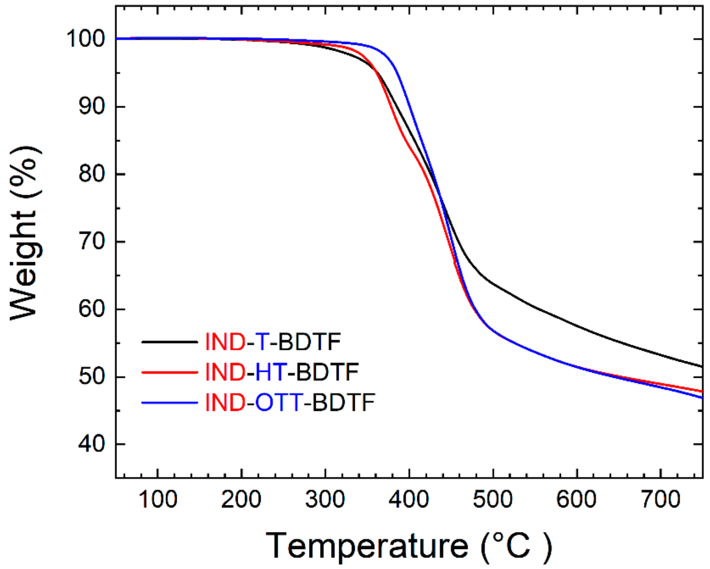

Figure S26. TGA thermograms of IND-T-BDTF, IND-HT-BDTF, and IND-OTT-BDTF.

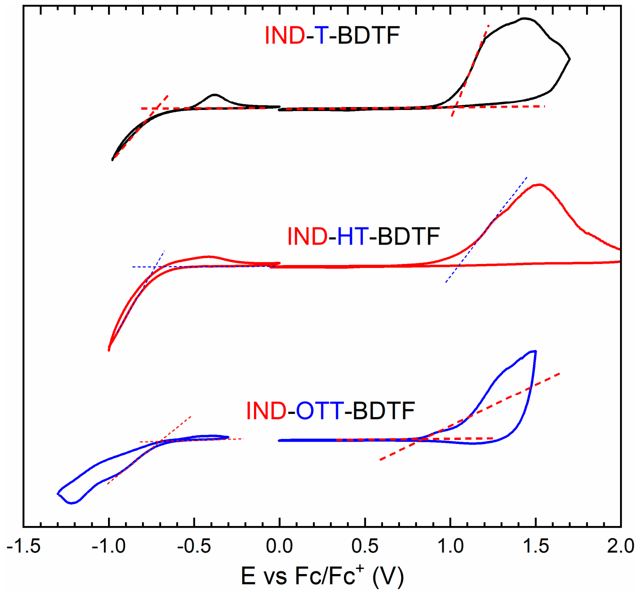

Figure S27. CV curves of (a) the reduction cycles and (b) the oxidation cycles of IND-T-BDTF, IND-HT-BDTF, and IND-OTT-BDTF.

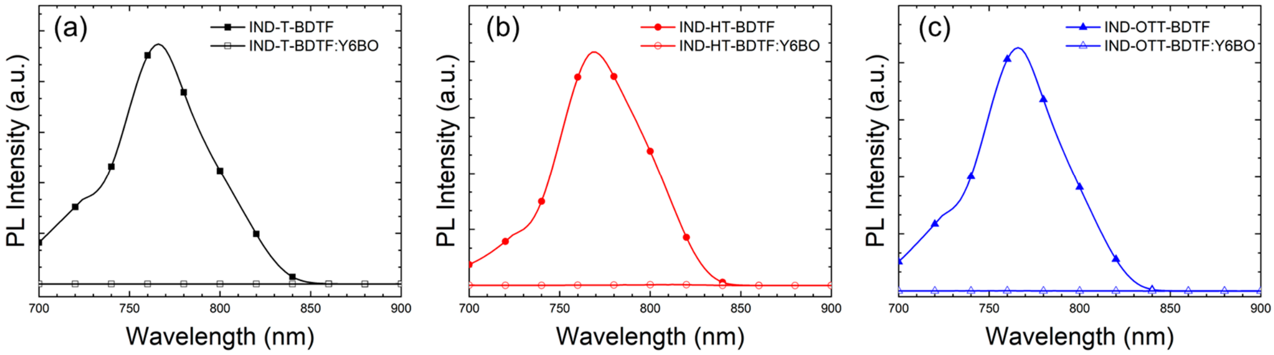

Figure S28. PL spectra of the polymers and their blend films with Y6BO acceptor: (a) IND-T-BDTF, (b) IND-HT-BDTF, and (c) IND-OTT-BDTF.

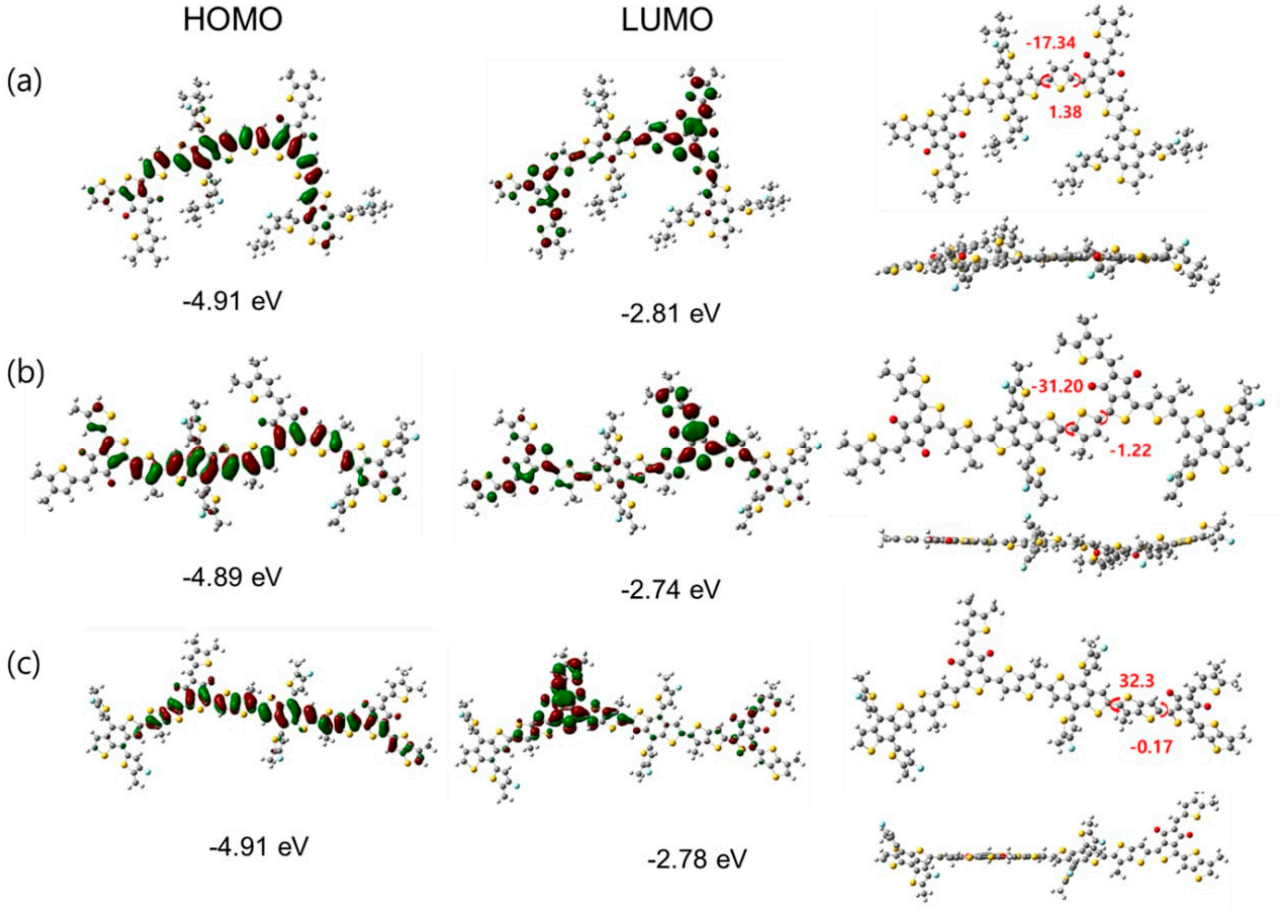

Figure S29. Frontier molecular orbitals of a two-repeating unit model compound at optimized geometry obtained from B3LYP/6-31G** level for (a) IND-T-BDTF, (b) IND-HT-BDTF, IND-OTT-BDTF.

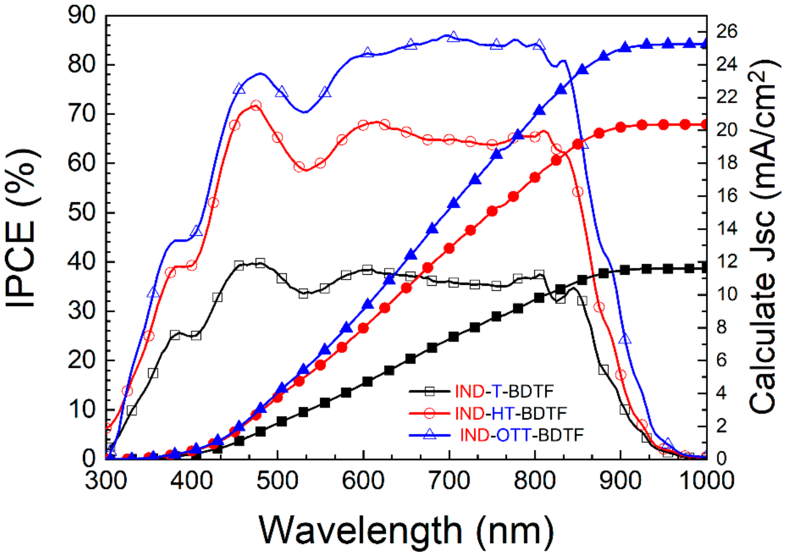

Figure S30. The IPCE spectra of the PSCs based on IND-T-BDTF, IND-HT-BDTF, and IND-OTT-BDTF.

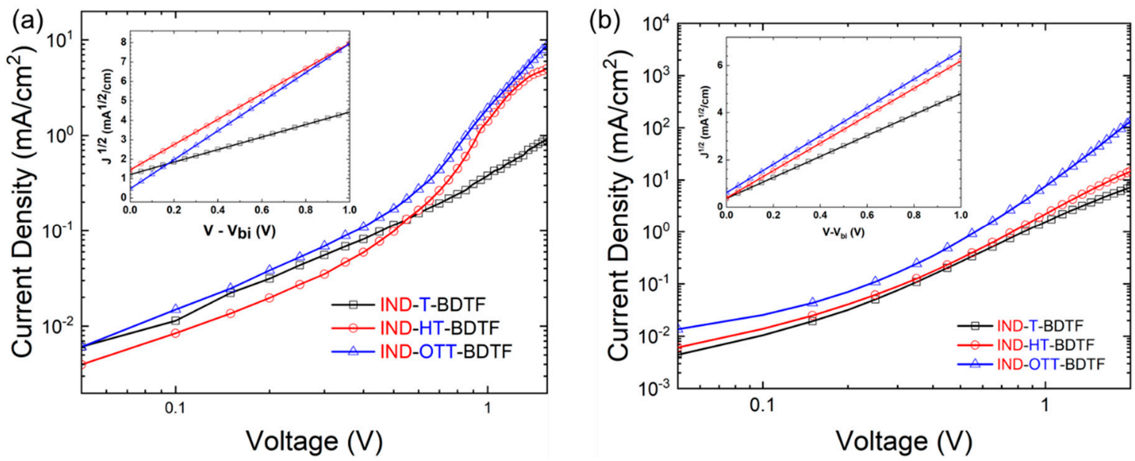

Figure S31. *J-V* curves of (a) hole- and (b) electron-only devices (inset: current density vs. voltage – built-in voltage (V_{bi}) curves with fitted lines.

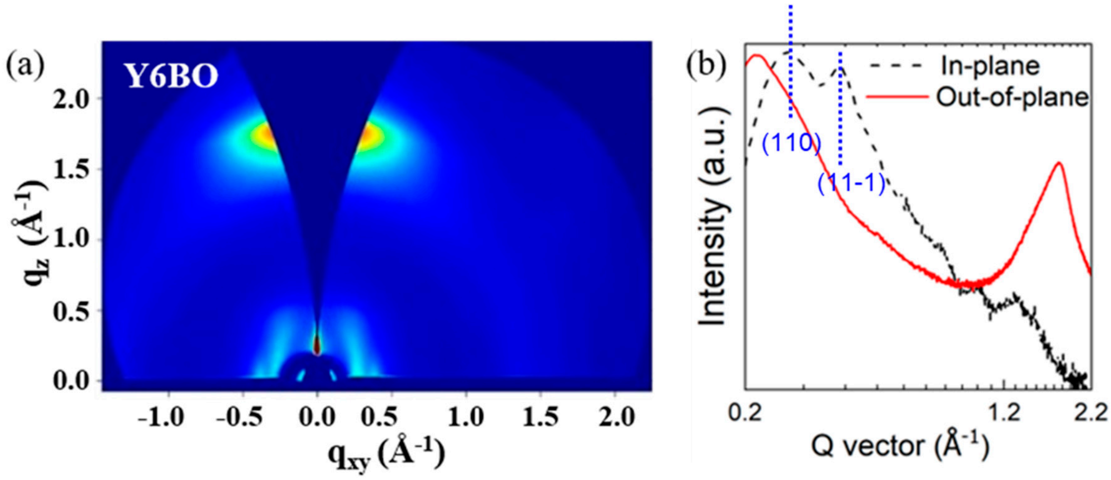

Figure S32. (a) GIWAXS image of pristine Y6BO film and (b) the corresponding line-cuts in the in-plane and out-of-plane direction.

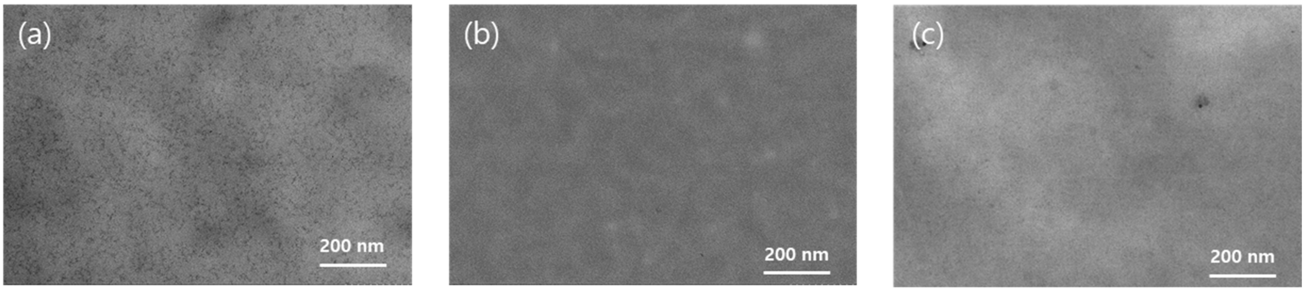

Figure S33. TEM images of the active layer based on (a) IND-T-BDTF, (b) IND-HT-BDTF, (c) IND-OTT-BDTF.

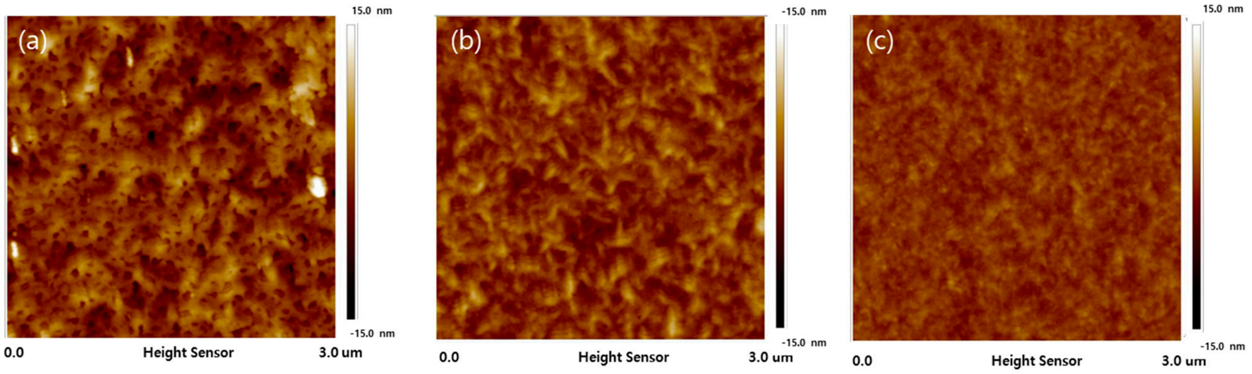

Figure S34. AFM images of the active layer based on (**a**) IND-T-BDTF, (**b**) IND-HT-BDTF, (**c**) IND-OTT-BDTF.

Table S1. The best photovoltaic parameters of the PSCs based on Y6BO. The average (10 devices are averaged) values for the photovoltaic parameters of each device are also provided in parentheses.

	**Blend Ratio**	**Thickness (nm)**	**J_{sc} (mA/cm^2)**	**V_{oc} (V)**	**FF (%)**	**PCE (%)**
PM6	1: 1.2	(90nm)	26.4 (26.2)	0.82 (0.82)	68.2 (68.2)	14.7 (14.6)
IND-T-BDTF	3 : 3	(100nm)	9.49 (9.23)	0.78 (0.78)	34.8 (34.75)	2.58 (2.50)
	3 : 4	(130nm)	11.85 (11.80)	0.80 (0.80)	33.5 (33.45)	3.18 (3.14)
	3 : 5	(146nm)	9.36 (8.96)	0.79 (0.79)	35.1 (35.25)	2.59 (2.49)
	3 : 6	(180nm)	8.95 (8.76)	0.79 (0.79)	34.8 (35.10)	2.46 (2.42)
	3 : 4	(138nm)	9.03 (8.97)	0.79 (0.78)	36.0 (36.07)	2.54 (2.52)
		(110nm)	10.07 (9.65)	0.79 (0.79)	35.5 (35.35)	2.82 (2.73)

		(98nm)	9.58 (9.29)	0.78 (0.78)	36.2 (36.30)	2.70 (2.63)
	3 : 3	(86nm)	18.48 (18.18)	0.85 (0.84)	37.90 (37.90)	5.92 (5.79)
	3 : 4	(118nm)	21.92 (21.75)	0.87 (0.87)	37.90 (37.68)	7.23 (7.11)
	3 : 5	(148nm)	17.98 (17.52)	0.83 (0.83)	39.95 (40.00)	5.98 (5.81)
IND-HT-BDTF	3 : 6	(194nm)	17.33 (16.74)	0.84 (0.83)	37.50 (37.65)	5.46 (5.25)
		(121nm)	21.13 (20.84)	0.86 (0.86)	36.15 (35.62)	6.57 (6.38)
	3 : 4	(108nm)	18.88 (18.69)	0.87 (0.86)	38.05 (38.87)	6.58 (6.38)
		(95nm)	18.97 (18.11)	0.86 (0.86)	40.70 (41.05)	6.64 (6.36)
	3 : 3	(105nm)	23.02 (22.42)	0.80 (0.80)	56.7 (56.23)	10.40 (10.07)
	3 : 4	(117nm)	26.37 (25.21)	0.79 (0.79)	56.2 (56.38)	11.69 (11.19)
	3 : 5	(132nm)	23.94 (23.34)	0.77 (0.77)	52.0 (51.48)	9.58 (9.25)
IND-OTT-BDTF	3 : 6	(148nm)	22.35 (22.35)	0.77 (0.77)	48.5 (50.85)	8.34 (8.29)
		(138nm)	23.8 (23.6)	0.79 (0.80)	57.9 (57.4)	10.90 (10.78)
	3: 4	(126nm)	23.98 (23.72)	0.79 (0.80)	59.8 (58.85)	11.30 (11.10)
		(102nm)	23.81 (23.62)	0.79 (0.79)	60.0 (59.63)	11.30 (11.18)

	(117nm)<br>80 ºC	24.20<br>(24.14)	0.80<br>(0.80)	54.2<br>(52.83)	10.50<br>(10.20)
	(117nm)<br>90 ºC	24.24<br>(23.93)	0.80<br>(0.79)	53.4<br>(53.87)	10.40<br>(10.23)
	(117nm)<br>110 ºC	25.24<br>(24.85)	0.78<br>(0.78)	54.2<br>(53.55)	10.70<br>(10.40)
	(117nm)<br>120 ºC	24.23<br>(23.75)	0.78<br>(0.78)	56.1<br>(56.35)	10.60<br>(10.43)
